# Do people who highly value happiness tend to ruminate?

**DOI:** 10.1007/s12144-022-04131-6

**Published:** 2023-01-13

**Authors:** Shigeyuki Takai, Akira Hasegawa, Jun Shigematsu, Tetsuya Yamamoto

**Affiliations:** 1 Fukude West Hospital, 22 Isshiki, 437-1216 Iwata-shi, Shizuoka, Japan; 2 Department of Psychology, International University of Health and Welfare, 4-1-26 Akasaka, Minato-ku, 107-8402 Tokyo, Japan; 3 Faculty of Humanities, University of Toyama, 3190, 930-0555 Gofuku, Toyama, Japan; 4 Graduate School of Technology, Industrial and Social Sciences, Tokushima University, 1-1, Minamijosanjima-cho, 770-8502 Tokushima, Japan

**Keywords:** Valuing happiness, Rumination, Depression, Stress, Culture, Self-construal

## Abstract

Previous studies have suggested that an extremely strong desire for happiness might ironically reduce a person’s well-being, particularly among Western people. According to the goal progress theory and the theory of valuing happiness, rumination might explain the relationship between valuing happiness and well-being. Based on these theoretical rationales, this study examined the following hypotheses: (1) valuing happiness is significantly associated with rumination, (2) people who experience low life stress have a stronger association between valuing happiness and rumination, and (3) people with more interdependent self-construal have a weaker association between valuing happiness and rumination. University students in Japan participated in a cross-sectional study (*N* = 350; Study 1) and a 4-weeks longitudinal study (*N* = 329; Study 2). They responded to a packet of questionnaires assessing valuing happiness, trait rumination, depressive symptoms, negative events, and interdependent self-construal. Consistent with our hypothesis, valuing happiness was concurrently and longitudinally associated with increased rumination after controlling for depressive symptoms. However, negative events did not moderate the association between valuing happiness and rumination. Furthermore, Study 1, but not Study 2, indicated that the association between valuing happiness and rumination was stronger among students with highly interdependent self-construal than those with less interdependent self-construal. The preset findings indicated that valuing happiness might be a factor that perpetuates rumination. More sophisticated evidence on the influence of valuing happiness on rumination can lead to effective psychotherapies for decreasing rumination and depression.

**Supplementary Information:**

The online version contains supplementary material available at 10.1007/s12144-022-04131-6.

The response styles theory (Nolen-Hoeksema, 1991), which proposes that the style of responding to depressed moods influences the course of depression, is one influential theory explaining the exacerbation and duration of depression. Previous studies have shown that rumination, among response styles indicated in this theory, is consistently related to depression. Rumination is defined as “behaviors and thoughts that focus one’s attention on one’s depressive symptoms and on the implications of these symptoms” (Nolen-Hoeksema, 1991, p. 569) and a form of repetitive negative thinking (Ehring & Watkins, 2008). Numerous longitudinal and experimental studies have indicated that rumination is a robust predictor of depressive symptoms and depressive disorders (for review, Nolen-Hoeksema et al., 2008). Studies have also suggested that rumination leads to harmful consequences such as increased negative thinking, interference with effective social problem solving and active instrumental behaviors, and decreased sensitivity to changing contingencies and contexts (for review, Watkins & Roberts, 2020).

Many theories explain the onset and persistence of rumination. One such theory is the goal progress theory (Martin et al., 2004), which assumes that a person who cannot attain goals tends to ruminate about the goals yet to be achieved. An experience-sampling study based on this theory demonstrated that low goal success and high goal importance were positively associated with concurrent state rumination (Moberly & Watkins, 2010). In addition, experimental studies have shown that cueing personal goal discrepancies caused more goal-focused rumination than cueing resolve goals (Roberts et al., 2013, 2020). Furthermore, frequent exposure to adverse events that prevent a person from attaining goals has prospective associations with the increase in rumination (Hamilton et al., 2015; Michl et al., 2013), although a nonsignificant association between these variables has also been demonstrated (Hasegawa et al., 2022).

According to the goal progress theory, people who overvalue happiness are prone to ruminate. Mauss et al. (2011) proposed a theory explaining the paradoxical effect of valuing happiness on decreased well-being. They suggested that people who highly value happiness tend to increase the standards of happiness they want to experience. As a result, they tend to be disappointed about their affective state, leading to decreased happiness. In addition, Mauss et al. (2011) proposed that because people attribute their unhappiness to their circumstances in adverse situations, even people who highly value happiness are not disappointed about their unhappiness when facing adverse conditions. In contrast, people that highly value happiness tend to be disappointed about their affective state in positive situations because they have reasons to feel happy.

Consistent with Mauss and colleagues’ theory, Mauss et al. (2011) showed that women who highly value happiness experience less well-being than those who value happiness less when facing low life stress, whereas this difference was not significant when facing high life stress. Mauss et al. (2011) also showed that women induced to value happiness experienced fewer positive emotions than women who underwent a task unrelated to valuing happiness when a happy mood was induced. On the other hand, such differences in positive emotions were not observed when a sad mood was induced. Subsequent studies conducted in Western countries have demonstrated that valuing happiness is related to increased loneliness (Mauss et al., 2012), depressive symptoms, and major depressive disorder (Bardeen & Fergus, 2020; Catalino et al., 2014; Fergus & Bardeen, 2016; Ford et al., 2014, 2015b; Gentzler et al., 2019; Kahriz et al., 2020), and increased risk and diagnosis of bipolar disorder (Ford et al., 2015b).

The goal progress theory and the theory of valuing happiness suggest that people who highly value happiness tend to experience a discrepancy between the ideal degree of happiness and actual happiness, leading to rumination about their disappointing affective state. In addition, the theory of valuing happiness suggests a stronger association between valuing happiness and rumination in people experiencing low than high life stress because the former cannot attribute their unhappiness to their adverse circumstances. Therefore, increased disappointment about the affective state by people that highly value happiness combined with low life stress might result in more rumination.

The above predictions are based on findings in Western countries. However, we might find different relationships between these variables in East Asian countries. Markus and Kitayama (1991) theorized that Westerners regard themselves as independent of others, whereas Asians regard themselves as interdependent within significant social units. Studies comparing Americans and Europeans with Japanese using questionnaires and behavioral measures assessing implicit attitudes, dispositional attribution, focused attention, and experience of disengaging emotions and personal forms of happiness have supported Markus and Kitayama’s theory (Kitayama et al., 2009; Na et al., 2020; Park et al., 2016). In addition, a recent study has suggested that cultural differences in self-construal have a neural basis (e.g., Yu et al., 2021).

Based on the theory by Markus and Kitayama (1991), Ford et al. (2015a) examined if valuing happiness, which was associated with decreased well-being among American undergraduate students, was associated with *increased* well-being among East Asian students (i.e., Japan and Taiwan). They suggested that people in collectivistic East Asian cultures define happiness as a positive feeling contingent on social engagement. Therefore, people in these countries who value happiness are likely to engage in social relationships such as prosocial behaviors and sharing intimate time with others. Such social engagement, in turn, can promote well-being. Therefore, valuing happiness might be associated with increased well-being among undergraduates in East Asia than in the US. Ford et al. (2015a) showed cross-cultural findings that supported their hypotheses. In addition, in line with the proposal by Ford et al. (2015a), valuing happiness was *positively* associated with specific variables related to well-being in Chinese people (Wong et al., 2020; Zhao et al., 2020) and Filipino adolescents (Datu et al., 2021).

These findings suggest that even people who highly value happiness and have a collectivistic view of the self might not frequently ruminate because they tend to engage in social relationships. Therefore, an interdependent self-construal, which is an individual difference in the collectivistic view of the self, might moderate the association between valuing happiness and rumination.

Despite theoretical rationales supporting the association between valuing happiness and rumination described above, previous studies have not examined this relationship. Therefore, a cross-sectional (Study 1) and a longitudinal study (Study 2) were conducted with university students in Japan to examine the association between valuing happiness and rumination.

The main purpose of this study was to examine the following hypotheses. We predicted that valuing happiness was significantly correlated with rumination because people who value happiness highly tend to experience a discrepancy between their ideal happiness and actual happiness, which would lead to ruminating about their disappointing affective condition. In addition, the increased disappointment about the affective state in people that highly value happiness and experience low life stress would lead to rumination because people in less adverse situations cannot attribute their unhappiness to their circumstances. Therefore, it was hypothesized that the fewer negative events students had experienced, the stronger the association between valuing happiness and rumination was. Furthermore, people that highly value happiness and have a more interdependent self-construal would not frequently ruminate because they may engage in social relationships. Therefore, this study examined the hypothesis that the more interpersonal a person’s self-construal was, the weaker the association between valuing happiness and rumination was. This study also examined these associations after controlling for the influence of depressive symptoms because studies have examined whether depressive symptoms confounded the association between rumination and other factors (see Zetsche et al., 2018).

## Study 1

Study 1, including the sample of non-selected university students in Japan, examined the concurrent association between valuing happiness and rumination and whether negative events and interpersonal self-construal moderated this association. This study also examined whether these relationships were significant even after controlling for depressive symptoms. Based on the theoretical rationales described in the Introduction, this study hypothesized that valuing happiness was significantly correlated with rumination, and this association is significant after controlling for depressive symptoms (Hypothesis 1). In addition, it was hypothesized that the fewer negative events the students had experienced, the stronger the association between valuing happiness and rumination was (Hypothesis 2). Furthermore, this study examined the hypothesis that the more interpersonal self-construal the students had, the weaker the association between valuing happiness and rumination was (Hypothesis 3).

Study 1 also explored the association between valuing happiness and brooding, the maladaptive aspect of rumination, and reflection, the adaptive or the less maladaptive aspect of rumination, in addition to the analyses predicting trait rumination in general. Brooding is the “passive comparison of one’s current situation with some unachieved standard,” and reflection is the “purposeful turning inward to engage in cognitive problem solving to alleviate one’s depressive symptoms” (Treynor et al., 2003, p. 256). Brooding and reflection are distinct subcomponents of rumination because brooding has a stronger association with depressive symptoms than reflection (e.g., Pearson et al., 2010; Schoofs et al., 2010; Treynor et al., 2003). Therefore, this study examined whether the associations with valuing happiness differed between brooding and reflection.

## Method

### Participants

Undergraduate students at Tokai Gakuin University and Hiroshima University in Japan (*N* = 390) participated in this study. Participants at Tokai Gakuin University were recruited in their classes. Students that agreed to participate in the study answered the questionnaires in the classrooms. Participants at Hiroshima University were recruited using snowball sampling. They responded to the questionnaires at a suitable time and place. Data of participants with missing values in any questionnaire, or inappropriate responses such as identical responses, were excluded from the analysis. The final sample comprised 350 students (203 men, 146 women, and 1 with unreported gender; mean age = 19.53 years, *SD* = 1.42; age range 18 to 34 years). All participants were Japanese except one Chinese and one German. Among the participants, 213 were first-year students, 63 second-year students, 68 third-year students, 3 fourth-year students, and 3 participants who did not report their year.

### Measures

***Valuing Happiness Scale*** (Mauss et al., 2011). This scale assesses individual differences in valuing happiness. The scale comprises seven items (e.g., “Feeling happy is extremely important to me.”). The responses to the scale are made on a 7-point rating scale ranging from 1 (*strongly disagree*) to 7 (*strongly agree*). The Japanese translation of the scale by Ford et al. (2015a) was used. The total score on the Valuing Happiness Scale was calculated. The scale has acceptable internal consistency (*α* = 0.78).

***Ruminative Responses Scale*** (RRS; Nolen-Hoeksema & Morrow, 1991**).** The RRS is designed to assess rumination. The respondents rate the 22 items of this scale using a 4-point scale ranging between 1 (*almost never*) and 4 (*almost always*). Treynor et al. (2003) suggested that depressive symptoms confound 12 RRS items. They conducted an exploratory factor analysis on the remaining ten items and extracted two factors, which they named brooding (e.g., “Thinking ‘Why do I have problems other people don’t have?’”), and reflection (e.g., “Analyze recent events to try and understand why you are depressed”). Brooding and reflection subscales have the advantage of comprising items seemingly unconfounded by depressive symptoms. However, they are limited by relatively weak internal consistency, which might weaken their power to predict depressive symptoms (see Hasegawa et al., 2018). In contrast, the total RRS score, which sums up the scores of all 22 items, has good internal consistency. The total RRS score has been used in previous studies, including recent intervention studies (e.g., Hoorelbeke et al., 2021; Hvenegaard et al., 2020; McIndoo et al., 2016) and longitudinal studies (e.g., de Klerk-Sluis et al., 2022; Ronold et al., 2020; Wolitzky-Taylor et al., 2021). The current study analyzed both brooding and reflection subscale scores and the total RRS score because these scores have been used in many previous studies.

Previous studies have reported the good psychometric properties of the RRS (Schoofs et al., 2010; Treynor et al., 2003). We used the Japanese version of the RRS (Hasegawa, 2013). Brooding and reflection subscale scores and the total RRS score were calculated. The alpha coefficients of the scale were 0.87, 0.77, and 0.95, respectively, for the brooding and reflection subscales and the overall RRS in this sample.

***Beck Depression Inventory-Second Edition*** (Beck et al., 1996). This scale is a well-validated self-report measure of depressive symptoms experienced in the past two weeks. The respondents answered 21 items using a scale anchored between 0 and 3. We used the Japanese version of the scale (Kojima & Furukawa, 2003). The total score on this scale was calculated. The internal consistency of this scale was good in our sample (*α* = 0.93).

***Scale of Life Events in Interpersonal and Achievement Domains*** (Takahira, 1998**).** This scale assesses negative and positive events that university students tend to experience. This scale comprises 30 negative event items (e.g., “I quarreled with a family member, friend, or romantic partner,” and “There were many tasks such as reports that should be come to grips with”) and 30 positive event items (e.g., “I have more friends to enjoy with,” and “I got good grades in my exams or reports”). Participants respond to the 60 items by indicating the frequency of encountering each event during the last three months. Participants respond to the scale using a 2-point scale anchored between 0 (*no*) and 1 (*yes*). The negative events subscale and the positive events subscale scores were calculated. The scale demonstrated good internal consistencies for negative (*α* = 0.91) and positive events (*α* = 0.89) subscales in this study.

***Independent and Interdependent Self-Construal Scale*** (Takata et al., 1996). This scale assesses individual differences in interdependent and independent self-construal. The scale comprises ten items assessing the interdependent self-construal (e.g., “I’m concerned about what other people think of me” and “It is important to stay in harmony with my peers”) and ten items assessing the independent self-construal (e.g., “I act as I believe even if the people around me have different ideas than I do” and “I always have my own opinion”).^1^ The two subscales demonstrated adequate internal consistency, test-retest reliability, and good construct validity in a study examining the correlations of interdependent and independent self-construal subscale scores with other related variables (Takata et al., 1996). In addition, consistent with the theory by Markus and Kitayama (1991), Japanese university students scored higher on the interdependent self-construal subscale and lower on the independent self-construal subscale than Australian and Canadian university students (Takata, 1999). The respondents answered the 20 items using a scale ranging from 1 (*not at all true of me*) to 7 (*extremely true of me*). Adequate internal consistency was demonstrated for the interdependent self-construal (*α* = 0.85) and the independent self-construal subscales (*α* = 0.81).

### Procedure

Study 1 was conducted from January to March 2021. COVID-19 spread in Japan after February 2020. Depending on its spread, the classes in Tokai Gakuin University were conducted online and face-to-face at different times. All classes in this university were conducted face-to-face in January 2021, when the survey was conducted at this university. In contrast, only seminars and practical training were conducted face-to-face at Hiroshima University, whereas other classes were held online until March 2021.

We explained the study to the students before they participated. Only students who agreed to participate in this study answered the questionnaires. The Ethics Committee of Tokai Gakuin University approved the studies described in this article.

### Statistical analysis

Descriptive statistics were identified, and the missing completely at random (MCAR) test was conducted using SPSS ver. 23 (IBM Corporation). Other analyses were conducted using Mplus 8.3 (Muthén & Muthén, 1998–2017). Pearson’s correlations among all variables were calculated. Multiple regression analyses with the maximum likelihood estimation method were conducted to examine the interaction of valuing happiness with negative events or interdependent self-construal on rumination. In addition, regression analyses were conducted with depressive symptoms as a covariate. Study 1 also conducted correlation analyses and multiple regression analyses using positive events and independent self-construal. However, these analyses were exploratory, and we have not further discussed their findings.

Little’s MCAR test (1988) using age and gender information and all the scale scores in the final sample yielded a nonsignificant chi-square value (*χ*^2^ (157) = 163.00, *p* = .355), suggesting the values were missing at random. Missing data were handled with multiple imputations using Bayesian analysis when conducting correlation and path analyses. Imputation was conducted using age, gender, and all items of the scales described in the Measures section, and 20 data sets were generated and used for analyses.

## Results

Table 1 displays descriptive statistics of each measure, and Table 2 shows the correlations between each measure. Valuing happiness had significant positive correlations with brooding, reflection, the total RRS score, negative and positive events, and interdependent and independent self-construal. In addition, the relationships of valuing happiness with brooding, reflection, and total RRS score remained significant even after controlling for the influences of depressive symptoms (*prs* = 0.26, 0.25, and 0.29, respectively, all *p*s < 0.001).

**Table 1:** Descriptive statistics of the measures used in Study 1

	*n*	*M*	*SD*	Range	Skewness	Kurtosis
Valuing happiness	342	30.79	7.22	7–49	–0.35	1.17
Brooding	345	10.39	4.06	5–20	0.39	–0.78
Reflection	345	8.88	3.46	5–20	0.83	0.11
Rumination total	344	43.49	15.41	22–86	0.45	–0.51
Depressive symptoms	333	12.81	10.81	0–63	1.07	1.17
Negative events	332	11.24	7.14	0–30	0.69	–0.10
Positive events	338	15.03	7.04	0–30	0.18	–0.61
Interdependent self-construal	341	47.98	9.19	14–69	–0.47	0.43
Independent self-construal	340	41.72	8.50	11–67	–0.24	1.11

**Table 2:** Correlations between variables in Study 1

	1	2	3	4	5	6	7	8
1. Valuing happiness								
2. Brooding	0.24							
3. Reflection	0.25	0.67						
4. Rumination total	0.26	0.94	0.82					
5. Depressive symptoms	0.06	0.59	0.40	0.62				
6. Negative events	0.21	0.28	0.35	0.33	0.39			
7. Positive events	0.28	–0.02	0.18	0.04	–0.08	0.56		
8. Interdependent self-construal	0.39	0.40	0.22	0.38	0.11	0.03	0.02	
9. Independent self-construal	0.16	–0.16	0.06	–0.10	–0.28	–0.04	0.19	0.06

Note: Absolute correlations of 0.11 or greater are significant at *p* < .05.

Separate multiple regression analyses were conducted to examine the main effect of valuing happiness and one of the hypothesized moderators (negative events or interdependent self-construal) and the interaction of valuing happiness with either moderator on the total RRS score. As shown in Table 3, the analysis with negative events as a moderator showed that valuing happiness and negative events had significant positive associations with rumination. However, the interaction between these two variables was not significant. The main effect of valuing happiness remained significant after controlling for depressive symptoms, whereas the significant association between negative events and rumination disappeared.

**Table 3:** Results of multiple regression analyses with rumination as a dependent variable in Study 1

	No covariate						Depressive symptoms as a covariate				
	*B*	*SE*	*β*	95%CI	*R* ^2^		*B*	*SE*	*β*	95%CI	*R* ^2^
Analysis 1: Negative events as a moderator					0.15						0.44
Valuing happiness	0.43	0.11	0.20***	[0.11, 0.30]			0.46	0.09	0.22***	[0.14, 0.30]	
Negative events	0.61	0.11	0.28***	[0.19, 0.38]			0.12	0.10	0.06	[–0.03, 0.15]	
Valuing happiness × Negative events	0.02	0.01	0.06	[–0.04, 0.16]			0.01	0.01	0.03	[–0.05, 0.11]	
Depressive symptoms							0.83	0.06	0.58***	[0.51, 0.65]	
Analysis 2: Interdependent self-construal as a moderator					0.18						0.50
Valuing happiness	0.27	0.11	0.13*	[0.03, 0.23]			0.26	0.09	0.12**	[0.04, 0.20]	
Interdependent self-construal	0.55	0.09	0.33***	[0.24, 0.43]			0.45	0.07	0.28***	[0.19, 0.36]	
Valuing happiness × Interdependent self-construal	0.03	0.01	0.15**	[0.05, 0.24]			0.02	0.01	0.10**	[0.03, 0.18]	
Depressive symptoms							0.82	0.06	0.57***	[0.51, 0.64]	
Analysis 3: Positive events as a moderator					0.07						0.43
Valuing happiness	0.59	0.11	0.28***	[0.18, 0.38]			0.48	0.09	0.23***	[0.15, 0.31]	
Positive events	–0.09	0.12	–0.04	[–0.15, 0.07]			0.05	0.09	0.02	[–0.06, 0.11]	
Valuing happiness × Positive events	0.01	0.02	0.04	[–0.06, 0.14]			0.01	0.01	0.05	[–0.04, 0.13]	
Depressive symptoms							0.87	0.06	0.61***	[0.54, 0.67]	
Analysis 4: Independent self-construal as a moderator					0.09						0.43
Valuing happiness	0.61	0.11	0.29***	[0.19, 0.39]			0.47	0.09	0.22***	[0.14, 0.30]	
Independent self-construal	–0.26	0.09	–0.15**	[–0.25, –0.04]			0.06	0.08	0.04	[–0.05, 0.12]	
Valuing happiness × Independent self-construal	–0.00	0.01	–0.01	[–0.12, 0.09]			–0.00	0.01	–0.02	[–0.10, 0.06]	
Depressive symptoms							0.88	0.06	0.61***	[0.54, 0.68]	

Note: Numbers in parentheses indicate 95% confidence intervals. * *p* < .05, ** *p* < .01, *** *p* < .001.

The analysis with interdependent self-construal as a moderator showed that the main effects of valuing happiness and interdependent self-construal and the interaction of these variables on rumination were significant (see Table 3). Simple slope analysis indicated that valuing happiness was not significantly associated with rumination (*β* = 0.00, *p* = .962) in participants with a less interdependent self-construal (i.e., *M* – 1*SD*). However, valuing happiness had a significant positive association with rumination (*β* = 0.25, *p* < .001) in participants with a high interdependent self-construal (i.e., *M* + 1*SD*). Moreover, all the main effects and the interaction were significant after controlling for depressive symptoms.

We also conducted regression analyses with positive events and independent self-construal as moderators (see Table 3). The analysis with positive events as a moderator showed that only valuing happiness had a significant positive association with rumination. Similar results were obtained after controlling for depressive symptoms. Furthermore, the analysis with independent self-construal as a moderator showed that valuing happiness had a significant positive association with rumination and independent self-construal had a significant negative association with rumination. However, the significant association between independent self-construal and rumination disappeared after controlling for depressive symptoms.

Finally, we conducted a supplementary analysis with brooding or reflection as the dependent variables. Results of the analysis using brooding were nearly identical to those using the total RRS score (see Table S1). The results of the analysis using reflection were slightly different from those using the total RRS score in terms of the significant association between negative events and reflection when depressive symptoms were a covariate. Nevertheless, the results associated with the hypotheses were very similar to those using the total RRS score (see Table S2).

## Discussion

Valuing happiness was positively correlated with rumination assessed by the total RRS score, which supported Hypothesis 1. In addition, this correlation was significant even after controlling for depressive symptoms. These results can be interpreted based on the goal progress theory (Martin et al., 2004) and the theory of valuing happiness (Mauss et al., 2011) as people who highly value happiness can experience a discrepancy between their ideal degree of happiness and actual happiness and frequently ruminate about their disappointing affective state.

However, negative events did not moderate the association between valuing happiness and rumination, which did not support Hypothesis 2, that people who experience fewer negative events have a stronger association between valuing happiness and rumination. Nevertheless, the main effect of negative events on rumination was significant, although it disappeared after controlling for depressive symptoms. The positive relationship between negative events and rumination is in line with the goal progress theory (Martin et al., 2004), suggesting that experiencing adverse events preventing a person from attaining goals can lead to rumination about discrepancies between goals and actual states. Therefore, the goal progress theory might better explain the influence of negative events on rumination than the theory of valuing happiness.

Moreover, there was a significant moderating effect of interdependent self-construal on the association between valuing happiness and rumination. However, interdependent self-construal *strengthened* this association, which was inconsistent with Hypothesis 3, which predicted that interdependent self-construal would *weaken* this association. The item content of the scale used in Study 1 might explain the opposite result of Hypothesis 3. Many items in the interdependent self-construal subscale reflect concerns about evaluation by others, such as “I’m concerned about what other people think of me” (see Footnote 1). Therefore, people who score high on the Valuing Happiness Scale and the interdependent self-construal subscale might want to avoid negative evaluations from others to be happy. Researchers have suggested that the avoidance goal might hinder progress towards goal achievement because these goals provide little concrete guidance on the steps necessary for goal achievement, making it difficult to achieve goals (Watkins & Roberts, 2020). Indeed, Thomsen et al. (2011) showed that trait rumination was positively correlated with avoidance goals.

Finally, the results using the brooding and reflection subscale scores as measures of rumination were approximately similar to those using the total RRS score. Valuing happiness was positively correlated with brooding and reflection subcomponents of rumination, and the interaction between valuing happiness and negative events on these two subcomponents was nonsignificant. Moreover, the interaction between valuing happiness and interdependent self-construal was significant (but the results of simple slope analysis did not support Hypothesis 3). Many studies have suggested that brooding is a maladaptive aspect of rumination that exacerbates depressive symptoms (for reviews, Nolen-Hoeksema et al., 2008; Watkins & Roberts, 2020), and reflection is an adaptive or less maladaptive aspect of rumination that is related to effective problem solving (Burwell & Shirk, 2007; Hasegawa et al., 2016). These findings suggest that the content of goals leading to brooding and reflection might differ. However, Study 1 indicated that valuing happiness might be a common factor leading to both subcomponents of rumination.

## Study 2

Study 1 indicated that people who value happiness to an extreme degree tend to ruminate frequently. However, Study 1, which was cross-sectional, could not determine the causality between valuing happiness and rumination. Therefore, a longitudinal study was conducted to examine the causality among variables.

We examined the following hypotheses in Study 2 based on the identical theoretical rationale as in Study 1. At first, valuing happiness at baseline is significantly associated with increased rumination assessed four weeks later after controlling for baseline rumination, which is significant even after controlling for baseline depressive symptoms (Hypothesis 1). In addition, students experiencing fewer negative events during the follow-up period show a stronger association between valuing happiness at baseline and subsequent rumination (Hypothesis 2). Furthermore, the association between valuing happiness at baseline and subsequent rumination is weak in students with a more interpersonal self-construal (Hypothesis 3). Similar to Study 1, Study 2 also analyzed the data after replacing rumination in general with brooding and reflection.

## Method

### Participants

Undergraduate and graduate students at Tokai Gakuin University, Tokushima University, and the University of Toyama in Japan participated in this study. Participants at Tokai Gakuin University were recruited in their classes, and those at the other universities were recruited with snowball sampling. Participants (*n* = 456) first responded to questionnaires in May and June of 2021 (Time 1). Students at Tokai Gakuin University who agreed to participate responded to the questionnaires in the classroom. The participants at the other universities responded to the questionnaires at a time and place suitable to them.

The participants (*n* = 475) took part in the second survey four weeks later (Time 2). Participants at Tokai Gakuin University responded in the same classes precisely 28 days after Time 1. The participants at Tokushima University and the University of Toyama were asked to return the questionnaires 28 days after Time 1. However, some participants at Tokushima University and all the participants at the University of Toyama failed to return the questionnaires in exactly 28 days.

We excluded the data of participants who did not participate at both time points, participants with missing data in any of the questionnaires, inappropriate responses such as identical responses, and those who did not return the questionnaires precisely 28 days after Time 1. The final sample comprised 329 students (188 men, 140 women, and 1 with unreported gender). The mean age of the final sample at Time 1 was 19.37 years (*SD* = 3.24), and the age range was 18 to 50 years. All the participants were Japanese. Among the participants, 152 were first-year students, 116 second-year students, 28 third-year students, 27 fourth-year students, 1 first-year master’s student, and 5 second-year master’s students.

### Measures

***Valuing Happiness Scale, Ruminative Responses Scale, and Beck Depression Inventory-Second Edition.*** The identical scales as in Study 1 were used to assess valuing happiness, trait rumination, and depressive symptoms in Study 2. The alpha coefficients of the scales at Time 1 were 0.79 for the Valuing Happiness Scale, 0.83, 0.79, and 0.94 for the brooding and reflection subscales and the overall RRS, and 0.93 for the Beck Depression Inventory-Second Edition, respectively. The alpha coefficients of the scales at Time 2 were 0.88, 0.81, and 0.96 for the brooding and reflection subscales, and the overall RRS, respectively.

***Scale of Life Events in Interpersonal and Achievement Domains and Independent and Interdependent Self-Construal Scale***. These scales were also identical to those used in Study 1. However, only the negative events subscale of the former and the interdependent self-construal subscale of the latter scales were used in Study 2 to reduce the participants’ burden. In Study 2, participants respond to the negative events items by indicating each events’ experiences during the last four weeks. Alpha coefficients of the interdependent self-construal subscale at Time 1 were 0.87, and that of the negative events subscale at Time 2 was 0.92.

### Procedure

Study 2 was conducted from March to July 2021. All classes in Tokai Gakuin University were conducted online during the two weeks of the follow-up period due to the spread of COVID-19, and were conducted face-to-face during the rest of the period. On the other hand, only seminars and practical training were conducted face-to-face at Tokushima University and the University of Toyama, whereas other classes were conducted online during the period of this study.

Similar to Study 1, only students who agreed to participate responded to the questionnaires. Participants wrote their birthdays and the last four digits of their mobile phone numbers in the spaces provided in the questionnaire packet. These numbers were used to match the survey data of the two time points.

### Statistical analysis

We conducted similar analyses in Study 2 as in Study 1. We have described only the results of variables used in the multiple regression analyses. The MCAR test was conducted using information about the age, gender, and all the scale scores at both time points. The final sample yielded a non-significant chi-square value (*χ*^2^ (253) = 284.86, *p* = .082), suggesting values were missing at random. The missing data were handled with multiple imputations using the identical procedures as Study 1. Imputation was conducted using age, gender, and all items of the scales described in the Measures section at both time points.^2^

## Results

Table 4 shows descriptive statistics, and Table 5 shows the correlations between measures. A multiple regression analysis was conducted to examine whether valuing happiness at baseline was associated with an increase in the total RRS score assessed four weeks later. As shown in Table 6, valuing happiness had a positive but nonsignificant association with subsequent rumination after controlling for baseline rumination. Moreover, valuing happiness at the baseline had a significant positive association with subsequent rumination after controlling for baseline depressive symptoms and rumination.

**Table 4:** Descriptive statistics of the measures in Study 2

	*n*	*M*	*SD*	Range	Skewness	Kurtosis
*Time 1*						
Valuing happiness	328	29.78	7.87	7–49	–0.24	–0.20
Brooding	326	10.76	4.00	5–20	0.22	–0.90
Reflection	328	8.85	3.48	5–19	0.90	–0.04
Rumination total	321	43.91	14.29	22–83	0.33	–0.66
Depressive symptoms	317	12.63	10.38	0–52	1.20	1.32
Interdependent self-construal	324	50.42	10.12	10–70	–0.76	1.35
*Time 2*						
Brooding	326	10.73	4.30	5–20	0.35	–0.88
Reflection	326	8.81	3.61	5–20	0.94	0.34
Rumination total	317	44.42	16.02	22–88	0.39	–0.70
Negative events	322	8.50	6.90	0–30	0.97	0.31

**Table 5:** Correlations between variables in Study 2

	1	2	3	4	5	6	7	8	9
*Time 1*									
1. Valuing happiness									
2. Brooding	0.32								
3. Reflection	0.23	0.61							
4. Rumination total	0.33	0.89	0.79						
5. Depressive symptoms	0.06	0.48	0.35	0.55					
6. Interdependent self-construal	0.39	0.48	0.30	0.48	0.31				
*Time 2*									
7. Brooding	0.27	0.64	0.46	0.64	0.47	0.41			
8. Reflection	0.26	0.42	0.60	0.52	0.32	0.24	0.67		
9. Rumination total	0.29	0.61	0.54	0.68	0.52	0.38	0.93	0.83	
10. Negative events	0.12	0.30	0.27	0.36	0.40	0.13	0.36	0.39	0.45

Note: Absolute correlations of 0.12 or greater are significant at *p* < .05.

**Table 6:** Results of multiple regression analyses with rumination at Time 2 as a dependent variable in Study 2

	No covariate						Depressive symptoms as a covariate				
	*B*	*SE*	*β*	95%CI	*R* ^2^		*B*	*SE*	*β*	95%CI	*R* ^2^
Analysis 1: No moderator					0.46						0.50
Valuing happiness at Time 1	0.16	0.09	0.08	[–0.01, 0.16]			0.22	0.09	0.11**	[0.03, 0.19]	
Rumination total at Time 1	0.73	0.05	0.65***	[0.58, 0.72]			0.57	0.06	0.51***	[0.42, 0.61]	
Depressive symptoms at Time 1							0.35	0.07	0.23***	[0.14, 0.32]	
Analysis 2: Negative events as a moderator					0.51						0.53
Valuing happiness at Time 1	0.16	0.08	0.08	[–0.00, 0.16]			0.21	0.08	0.10*	[0.02, 0.18]	
Rumination total at Time 1	0.63	0.05	0.57***	[0.49, 0.64]			0.54	0.06	0.48***	[0.39, 0.57]	
Negative events at Time 2	0.54	0.10	0.23***	[0.15, 0.32]			0.45	0.10	0.19***	[0.11, 0.28]	
Valuing happiness at Time 1 × Negative events at Time 2	–0.01	0.01	–0.02	[–0.10, 0.05]			–0.01	0.01	–0.03	[–0.10, 0.05]	
Depressive symptoms at Time 1							0.27	0.07	0.18***	[0.08, 0.27]	
Analysis 3: Interdependent self-construal as a moderator					0.47						0.51
Valuing happiness at Time 1	0.14	0.09	0.07	[–0.02, 0.15]			0.21	0.09	0.11*	[0.02, 0.19]	
Rumination total at Time 1	0.70	0.05	0.62***	[0.55, 0.70]			0.56	0.06	0.50***	[0.40, 0.59]	
Interdependent self-construal at Time 1	0.11	0.08	0.07	[–0.03, 0.16]			0.07	0.07	0.04	[–0.05, 0.14]	
Valuing happiness at Time 1 × Interdependent self-construal at Time 1	0.01	0.01	0.07	[–0.01, 0.15]			0.01	0.01	0.07	[–0.01, 0.15]	
Depressive symptoms at Time 1							0.35	0.07	0.23***	[0.14, 0.32]	

Note: Numbers in parentheses indicate 95% confidence intervals. * *p* < .05, ** *p* < .01, *** *p* < .001.

Separate regression analyses were also conducted to examine the interaction of valuing happiness at baseline with either moderator (i.e., negative events experienced during the follow-up period or interdependent self-construal at baseline) on rumination four weeks later. As shown in Table 6, the analysis with negative events during the follow-up period as a moderator showed that negative events had a significant positive association with subsequent rumination, which was not the case for valuing happiness. Moreover, the interaction of valuing happiness with negative events on subsequent rumination was not significant. An analysis controlling for baseline depressive symptoms showed that valuing happiness at baseline and negative events during the follow-up period had significant positive associations with subsequent rumination. However, the interaction between these two independent variables was not significant.

An analysis using interdependent self-construal at Time 1 as a moderator showed that neither valuing happiness nor interdependent self-construal at baseline was related to subsequent rumination. The interaction between the two independent variables on subsequent rumination was not significant.^3^ Furthermore, valuing happiness at baseline was significantly associated with increased rumination after controlling for baseline depressive symptoms and rumination. Neither the main effect of interdependent self-construal at baseline nor the interaction between valuing happiness and interdependent self-construal on subsequent rumination was significant (see Table 6).

Results of analyses that replaced the total RRS score with the brooding subscale score were mainly similar to those using the total RRS score, except for the significant positive association between interdependent self-construal at baseline and subsequent brooding when depressive symptoms were not a covariate (see Table S3). On the other hand, results of using the reflection subscale score as the rumination measure were somewhat different from those using the total RRS score, with the results indicating that valuing happiness had a significant positive association with subsequent reflection when baseline depressive symptoms were not used as a covariate (see Table S4).

## Discussion

Study 2 demonstrated that valuing happiness at baseline had a positive but nonsignificant association with subsequent rumination after controlling for baseline rumination. This longitudinal association was significant after controlling for baseline depressive symptoms and rumination. These findings partially supported Hypothesis 1. The predictive power of valuing happiness on future rumination was slightly more robust with baseline depressive symptoms as a covariate than without it. This result might suggest that valuing happiness can lead to ruminative thoughts with less negative valence, which are less related to depressive symptoms. Consistent with this possibility, the analyses using RRS subscale scores showed that valuing happiness was related to subsequent reflection even when baseline depressive symptoms were not controlled, whereas valuing happiness was not related to subsequent brooding (see Table S3 and S4).

Study 2 indicated that the interaction of valuing happiness with negative events experienced during the follow-up period on subsequent rumination was not significant, but the main effect of negative events was significant. These findings were inconsistent with Hypothesis 2. In addition, the interaction of valuing happiness with interpersonal self-construal on subsequent rumination was not significant, which was inconsistent with Hypothesis 3. The nonsignificant interaction of valuing happiness with interpersonal self-construal might be due to the relatively low statistical power of the study (see Footnote 3). We have further discussed this result in the General Discussion.

## General discussion

The findings of the two studies generally supported the assumption that valuing happiness is one factor leading to rumination. Valuing happiness had a concurrent positive correlation with rumination, which was significant even after controlling for cross-sectionally assessed depressive symptoms (Study 1). Furthermore, valuing happiness was associated with an increase in subsequent rumination, but only when baseline depressive symptoms were statistically controlled (Study 2). Considering both the goal progress theory (Martin et al., 2004) and the theory of valuing happiness (Mauss et al., 2011), people who strongly value happiness experience a discrepancy between the ideal degree of happiness and actual happiness, which leads to rumination about their disappointing affective states. Although it is necessary to further examine the validity of this hypothesis through more detailed studies, valuing happiness could be one cause of rumination.

On the other hand, the results on the interactions of valuing happiness with either negative events or interpersonal self-construal were not consistent with the hypotheses of this study. In particular, Study 1 showed that valuing happiness was more strongly associated with rumination among university students with high interpersonal self-construal than with low interpersonal self-construal, which was the opposite of Hypothesis 3. Future studies should reexamine this finding using the following strategies. Firstly, although the longitudinal results of Study 2 examining the interaction of valuing happiness with interpersonal self-construal on subsequent rumination indicated a similar trend to Study 1, the result was not significant, probably due to the insufficient sample size (see Footnote 3). Therefore, it is necessary to reexamine this interaction on subsequent rumination in a longitudinal study with a larger sample. In addition, the relationship between valuing happiness and rumination should be compared between East Asian people, who have a predominantly collectivistic culture, and Western people, who have an essentially individualistic culture (Kitayama et al., 2009; Na et al., 2020; Park et al., 2016; Yu et al., 2021). Based on Ford et al. (2015a), we predict that valuing happiness is more strongly associated with rumination in Western countries than in East Asian countries. Such a cross-cultural study might indicate the possible effect of cultural construal of the self on the association between valuing happiness and rumination.

This research examined associations between identical variables in separate cross-sectional and longitudinal studies, which increased the robustness of the findings. These findings consistently suggested that valuing happiness is associated with increased rumination. The present study integrated three influential theories explaining several psychopathological symptoms and well-being, including the response styles theory (Nolen-Hoeksema, 1991; Nolen-Hoeksema et al., 2008), the goal progress theory (Martin et al., 2004), and the theory of valuing happiness (Mauss et al., 2011). Moreover, in examining the causes and correlates of rumination, this study investigated negative events, which are stress research variables, and interdependent self-construal, a cultural psychology variable.

This study has some limitations other than those described above. The study included only undergraduate and graduate students in Japan. Therefore, it is necessary to reexamine the present results in other age groups and clinical samples. In addition, all the findings of this study are correlational, and all the data were obtained using self-report measures. Therefore, it is necessary to conduct correlational studies assessing specific variables by using structured interviews or experimental studies that manipulate valuing happiness using the methods adopted by Mauss et al. (2011). Finally, this study was conducted during the COVID-19 pandemic when university students experienced various restrictions on their behaviors. Therefore, a replication study should be conducted after the pandemic.

The present findings suggest that interventions focusing on reducing the intense desire for happiness might be efficacious in alleviating chronic rumination. Current psychotherapies might decrease trait rumination at least partly by reducing the intense desire for happiness. For example, strategies to modify the goal of changing emotions to the goal of changing behaviors and the environment can help improve trait rumination. Consistent with this notion, behavioral activation focusing on facilitating approach behaviors and increasing response-contingent positive reinforcement effectively decreases trait rumination in depressed university students (McIndoo et al., 2016). In addition, the present findings suggest that mindfulness meditation focusing on developing skills for temporarily abandoning all goals might be effective for decreasing rumination driven by valuing happiness. Indeed, meta-analyses have shown that mindfulness-based interventions effectively reduce rumination (Perestelo-Perez et al., 2017) and repetitive negative thinking in general (Spinhoven et al., 2018). It is interesting to examine whether valuing happiness is a mediator of these two therapies’ effects on rumination. Further evidence on the influence of valuing happiness on rumination could help us develop more effective psychotherapies for decreasing rumination and depression.

## Electronic supplementary material

Below is the link to the electronic supplementary material.

Supplementary Material 1

## Data Availability

This study’s dataset can be found at the Open Science Framework [https://osf.io/k3hnw/].

## Footnotes

1 Takata et al. (1996) suggested that interdependent self-construal and the independent self-construal subscales can be further subdivided into two subscales. Specifically, the former can be subdivided into the evaluation apprehension subscale and the dependency subscale, and the latter can be subdivided into the dogmatism subscale and the individuality subscale. However, this study calculated and analyzed the interdependent self-construal subscale and independent self-construal subscale scores for brevity.

2 Imputation was conducted using the data of the four scales described in the Measures section at the two time points. However, we have displayed only the results related to the hypotheses in the Results section.

3 The interaction of valuing happiness with interpersonal self-construal on subsequent rumination was marginally significant (*p* = .089). We examined the simple slope of valuing happiness on subsequent rumination when participants’ interpersonal self-construal scores were high and low to determine whether the identical trends could be observed as Study 1 in Study 2. Results indicated that similar to Study 1, valuing happiness was not significantly associated with rumination (*β* = 0.01, *p* = .867) when participants were less interdependent (i.e., *M* – 1*SD*), but valuing happiness had a significant positive association with rumination (*β* = 0.12, *p* = .026) when participants were more interdependent (i.e., *M* + 1*SD*). The similar trends were shown even after controlling for baseline depressive symptoms (low interpersonal self-construal: *β* = 0.05, *p* = .389; high interpersonal self-construal: *β* = 0.16, *p* = .003). The nonsignificant interaction of valuing happiness with interpersonal self-construal on subsequent rumination might be due to the low statistical power because the results of simple slop analyses were very similar to Study 1.
