# Supplementary material for: Do people who highly value happiness tend to ruminate?
Source: Curr Psychol. 2023 Jan 13:1–13. Online ahead of print. doi: 10.1007/s12144-022-04131-6 (PMC9837463; doi:10.1007/s12144-022-04131-6)
Supplement: Supplementary file 1 — Supplementary Material 1 [file 12144_2022_4131_MOESM1_ESM.docx]

**Table S1.** Results of multiple regression analyses with brooding as a dependent variable in Study 1

|  | No covariate | | | | | | | | |  | Depressive symptoms as a covariate | | | | | | | | |
| --- | --- | --- | --- | --- | --- | --- | --- | --- | --- | --- | --- | --- | --- | --- | --- | --- | --- | --- | --- |
|  | *B* | | *SE* | | *β* | | 95%CI | *R*^2^ | |  | *B* | | *SE* | | *β* | | 95%CI | *R*^2^ | |
| Analysis 1: Negative events as a moderator |  |  |  |  |  |  |  | .11 |  |  |  |  |  |  |  |  |  | .40 |  |
| Valuing happiness | 0.11 |  | 0.03 |  | .19 | *** | [.09, .29] |  |  |  | 0.12 |  | 0.02 |  | .21 | *** | [.13, .29] |  |  |
| Negative events | 0.13 |  | 0.03 |  | .24 | *** | [.14, .34] |  |  |  | 0.01 |  | 0.03 |  | .01 |  | [–.08, .10] |  |  |
| Valuing happiness × Negative events | 0.00 |  | 0.00 |  | .02 |  | [–.08, .12] |  |  |  | –0.00 |  | 0.00 |  | –.01 |  | [–.09, .08] |  |  |
| Depressive symptoms | – | | – | | – | | – |  |  |  | 0.22 |  | 0.02 |  | .58 | *** | [.50, .65] |  |  |
|  |  |  |  |  |  |  |  |  |  |  |  |  |  |  |  |  |  |  |  |
| Analysis 2: Interdependent self-construal |  |  |  |  |  |  |  | .19 |  |  |  |  |  |  |  |  |  | .48 |  |
| Valuing happiness | 0.05 |  | 0.03 |  | .10 |  | [–.01, .20] |  |  |  | 0.05 |  | 0.02 |  | 0.09 | * | [.01, .18] |  |  |
| Interdependent self-construal | 0.16 |  | 0.02 |  | .36 | *** | [.27, .46] |  |  |  | 0.13 |  | 0.02 |  | 0.31 | *** | [.22, .39] |  |  |
| Valuing happiness × Interdependent self-construal | 0.01 |  | 0.00 |  | .14 | ** | [.04, .23] |  |  |  | 0.01 |  | 0.00 |  | 0.09 | * | [.01, .17] |  |  |
| Depressive symptoms | – | | – | | – | | – |  |  |  | 0.21 |  | 0.02 |  | 0.55 | *** | [.48, .62] |  |  |
|  |  |  |  |  |  |  |  |  |  |  |  |  |  |  |  |  |  |  |  |
| Analysis 3: Positive events as a moderator |  |  |  |  |  |  |  | .07 |  |  |  |  |  |  |  |  |  | .40 |  |
| Valuing happiness | 0.15 |  | 0.03 |  | .27 | *** | [.17, .37] |  |  |  | 0.12 |  | 0.02 |  | .22 | *** | [.14, .31] |  |  |
| Positive events | –0.05 |  | 0.03 |  | –.09 |  | [–.20, .02] |  |  |  | –0.02 |  | 0.03 |  | –.03 |  | [–.12, .06] |  |  |
| Valuing happiness × Positive events | 0.00 |  | 0.00 |  | .01 |  | [–.10, .11] |  |  |  | 0.00 |  | 0.00 |  | .01 |  | [–.07, .09] |  |  |
| Depressive symptoms | – | | – | | – | | – |  |  |  | 0.22 |  | 0.02 |  | .58 | *** | [.51, .65] |  |  |
|  |  |  |  |  |  |  |  |  |  |  |  |  |  |  |  |  |  |  |  |
| Analysis 4: Independent self-construal |  |  |  |  |  |  |  | .10 |  |  |  |  |  |  |  |  |  | .40 |  |
| Valuing happiness | 0.15 |  | 0.03 |  | .28 | *** | [.18, .37] |  |  |  | 0.12 |  | 0.02 |  | .22 | *** | [.13, .30] |  |  |
| Independent self-construal | –0.10 |  | 0.02 |  | –.20 | *** | [–.30, –.10] |  |  |  | –0.02 |  | 0.02 |  | –.03 |  | [–.12, .05] |  |  |
| Valuing happiness × Independent self-construal | –0.00 |  | 0.00 |  | –.01 |  | [–.11, .09] |  |  |  | –0.00 |  | 0.00 |  | –.02 |  | [–.10, .06] |  |  |
| Depressive symptoms | – | | – | | – | | – |  |  |  | 0.22 |  | 0.02 |  | .57 | *** | [.50, .64] |  |  |
| Note: Numbers in parentheses indicate 95% confidence intervals. * *p* < .05, ** *p* < .01, *** *p* < .001. | | | | | | | | | | | | | | | | | | | |

**Table S2.** Results of multiple regression analyses with reflection as a dependent variable in Study 1

|  | No covariate | | | | | | | | |  | Depressive symptoms as a covariate | | | | | | | | |
| --- | --- | --- | --- | --- | --- | --- | --- | --- | --- | --- | --- | --- | --- | --- | --- | --- | --- | --- | --- |
|  | *B* | | *SE* | | *β* | | 95%CI | *R*^2^ | |  | *B* | | *SE* | | *β* | | 95%CI | *R*^2^ | |
| Analysis 1: Negative events as a moderator |  |  |  |  |  |  |  | .16 |  |  |  |  |  |  |  |  |  | .24 |  |
| Valuing happiness | 0.09 |  | 0.02 |  | .19 | *** | [.09, .29] |  |  |  | 0.09 |  | 0.02 |  | .20 | *** | [.10, .29] |  |  |
| Negative events | 0.15 |  | 0.03 |  | .31 | *** | [.21, .40] |  |  |  | 0.09 |  | 0.03 |  | .18 | *** | [.08, .28] |  |  |
| Valuing happiness × Negative events | 0.00 |  | 0.00 |  | .06 |  | [–.04, .16] |  |  |  | 0.00 |  | 0.00 |  | .05 |  | [–.04, .14] |  |  |
| Depressive symptoms | – | | – | | – | | – |  |  |  | 0.10 |  | 0.02 |  | .32 | *** | [.22, .41] |  |  |
|  |  |  |  |  |  |  |  |  |  |  |  |  |  |  |  |  |  |  |  |
| Analysis 2: Interdependent self-construal |  |  |  |  |  |  |  | .11 |  |  |  |  |  |  |  |  |  | .24 |  |
| Valuing happiness | 0.09 |  | 0.03 |  | .19 | ** | [.08, .30] |  |  |  | 0.09 |  | 0.02 |  | .19 | *** | [.09, .29] |  |  |
| Interdependent self-construal | 0.06 |  | 0.02 |  | .15 | ** | [.05, .26] |  |  |  | 0.04 |  | 0.02 |  | .12 | * | [.01, .22] |  |  |
| Valuing happiness × Interdependent self-construal | 0.01 |  | 0.00 |  | .15 | ** | [.05, .25] |  |  |  | 0.01 |  | 0.00 |  | .12 | * | [.03, .22] |  |  |
| Depressive symptoms | – | | – | | – | | – |  |  |  | 0.12 |  | 0.02 |  | .37 | *** | [.28, .46] |  |  |
|  |  |  |  |  |  |  |  |  |  |  |  |  |  |  |  |  |  |  |  |
| Analysis 3: Positive events as a moderator |  |  |  |  |  |  |  | .08 |  |  |  |  |  |  |  |  |  | .24 |  |
| Valuing happiness | 0.11 |  | 0.03 |  | .23 | *** | [.12, .33] |  |  |  | 0.09 |  | 0.02 |  | .19 | *** | [.10, .29] |  |  |
| Positive events | 0.06 |  | 0.03 |  | .12 | * | [.01, .22] |  |  |  | 0.08 |  | 0.02 |  | .16 | ** | [.06, .26] |  |  |
| Valuing happiness × Positive events | 0.00 |  | 0.00 |  | .05 |  | [–.05, .15] |  |  |  | 0.00 |  | 0.00 |  | .05 |  | [–.04, .15] |  |  |
| Depressive symptoms | – | | – | | – | | – |  |  |  | 0.13 |  | 0.02 |  | .40 | *** | [.32, .49] |  |  |
|  |  |  |  |  |  |  |  |  |  |  |  |  |  |  |  |  |  |  |  |
| Analysis 4: Independent self-construal |  |  |  |  |  |  |  | .06 |  |  |  |  |  |  |  |  |  | .23 |  |
| Valuing happiness | 0.12 |  | 0.03 |  | .25 | *** | [.15, .35] |  |  |  | 0.10 |  | 0.02 |  | .21 | *** | [.11, .30] |  |  |
| Independent self-construal | 0.01 |  | 0.02 |  | .02 |  | [–.09, .12] |  |  |  | 0.06 |  | 0.02 |  | .14 | ** | [.05, .24] |  |  |
| Valuing happiness × Independent self-construal | 0.00 |  | 0.00 |  | .00 |  | [–.10, .11] |  |  |  | 0.00 |  | 0.00 |  | –.00 |  | [–.10, .09] |  |  |
| Depressive symptoms | – | | – | | – | | – |  |  |  | 0.14 |  | 0.02 |  | .43 | *** | [.34, .52] |  |  |
| Note: Numbers in parentheses indicate 95% confidence intervals. * *p* < .05, ** *p* < .01, *** *p* < .001. | | | | | | | | | | | | | | | | | | | |

**Table S3.** Results of multiple regression analyses with brooding at Time 2 as a dependent variable in Study 2

|  | No covariate | | | | | | | | |  | Depressive symptoms as a covariate | | | | | | | | |
| --- | --- | --- | --- | --- | --- | --- | --- | --- | --- | --- | --- | --- | --- | --- | --- | --- | --- | --- | --- |
|  | *B* | | *SE* | | *β* | | 95%CI | *R*^2^ | |  | *B* | | *SE* | | *β* | | 95%CI | *R*^2^ | |
| Analysis 1: No moderator | | | | | | |  | .41 |  |  |  |  |  |  |  |  |  | .45 |  |
| Valuing happiness at Time 1 | 0.04 |  | 0.02 |  | 0.07 |  | [–.02, .16] |  |  |  | 0.05 |  | 0.02 |  | .10 | * | [.01, .18] |  |  |
| Brooding at Time 1 | 0.66 |  | 0.05 |  | 0.62 | *** | [.55, .69] |  |  |  | 0.53 |  | 0.05 |  | .50 | *** | [.41, .59] |  |  |
| Depressive symptoms at Time 1 | – | | – | | – | | – |  |  |  | 0.10 |  | 0.02 |  | .23 | *** | [.14, .32] |  |  |
|  |  |  |  |  |  |  |  |  |  |  |  |  |  |  |  |  |  |  |  |
| Analysis 2: Negative events as a moderator | | | | | | |  | .44 |  |  |  |  |  |  |  |  |  | .47 |  |
| Valuing happiness at Time 1 | 0.04 |  | 0.02 |  | .07 |  | [–.02, .15] |  |  |  | 0.05 |  | 0.02 |  | .09 | * | [.00, .17] |  |  |
| Brooding at Time 1 | 0.60 |  | 0.05 |  | .56 | *** | [.49, .64] |  |  |  | 0.52 |  | 0.05 |  | .48 | *** | [.40, .57] |  |  |
| Negative events at Time 2 | 0.11 |  | 0.03 |  | .18 | *** | [.10, .27] |  |  |  | 0.08 |  | 0.03 |  | .13 | ** | [.04, .22] |  |  |
| Valuing happiness at Time 1 × Negative events at Time 2 | 0.00 |  | 0.00 |  | .01 |  | [–.07, .09] |  |  |  | 0.00 |  | 0.00 |  | .00 |  | [–.08, .08] |  |  |
| Depressive symptoms at Time 1 | – | | – | | – | | – |  |  |  | 0.08 |  | 0.02 |  | .19 | *** | [09, .28] |  |  |
|  |  |  |  |  |  |  |  |  |  |  |  |  |  |  |  |  |  |  |  |
| Analysis 3: Interdependent self-construal as a moderator | | | | | | |  | .43 |  |  |  |  |  |  |  |  |  | .46 |  |
| Valuing happiness at Time 1 | 0.02 |  | 0.03 |  | 0.04 |  | [–.05, .13] |  |  |  | 0.04 |  | 0.03 |  | .07 |  | [–.02, .16] |  |  |
| Brooding at Time 1 | 0.60 |  | 0.05 |  | 0.56 | *** | [.48, .65] |  |  |  | 0.50 |  | 0.06 |  | .47 | *** | [.37, .56] |  |  |
| Interdependent self-construal at Time 1 | 0.05 |  | 0.02 |  | 0.13 | * | [.03, .23] |  |  |  | 0.04 |  | 0.02 |  | .10 |  | [–.00, .19] |  |  |
| Valuing happiness at Time 1 × Interdependent self-construal at Time 1 | 0.00 |  | 0.00 |  | 0.06 |  | [–.03, .14] |  |  |  | 0.00 |  | 0.00 |  | .06 |  | [–.03, .14] |  |  |
| Depressive symptoms at Time 1 | – | | – | | – | | – |  |  |  | 0.09 |  | 0.02 |  | .22 | *** | [.13, .31] |  |  |
| Note: Numbers in parentheses indicate 95% confidence intervals. * *p* < .05, ** *p* < .01, *** *p* < .001. | | | | | | | | | | | | | | | | | | | |

**Table S4.** Results of multiple regression analyses with reflection at Time 2 as a dependent variable in Study 2

|  | No covariate | | | | | | | | |  | Depressive symptoms as a covariate | | | | | | | | |
| --- | --- | --- | --- | --- | --- | --- | --- | --- | --- | --- | --- | --- | --- | --- | --- | --- | --- | --- | --- |
|  | *B* | | *SE* | | *β* | | 95%CI | *R*^2^ | |  | *B* | | *SE* | | *β* | | 95%CI | *R*^2^ | |
| Analysis 1: No moderator | | | | | | |  | .37 |  |  |  |  |  |  |  |  |  | .39 |  |
| Valuing happiness at Time 1 | 0.06 |  | 0.02 |  | .13 | ** | [.04, .22] |  |  |  | 0.06 |  | 0.02 |  | .14 | ** | [.05, .22] |  |  |
| Reflection at Time 1 | 0.58 |  | 0.05 |  | .56 | *** | [.49, .64] |  |  |  | 0.54 |  | 0.05 |  | .52 | *** | [.44, .60] |  |  |
| Depressive symptoms at Time 1 | – | | – | | – | | – |  |  |  | 0.05 |  | 0.02 |  | .13 | ** | [.04, .23] |  |  |
|  |  |  |  |  |  |  |  |  |  |  |  |  |  |  |  |  |  |  |  |
| Analysis 2: Negative events as a moderator | | | | | | |  | .42 |  |  |  |  |  |  |  |  |  | .43 |  |
| Valuing happiness at Time 1 | 0.05 |  | 0.02 |  | .12 | ** | [.03, .20] |  |  |  | 0.06 |  | 0.02 |  | .12 | ** | [.04, .21] |  |  |
| Reflection at Time 1 | 0.52 |  | 0.05 |  | .50 | *** | [.43, .58] |  |  |  | 0.51 |  | 0.05 |  | .49 | *** | [.41, .57] |  |  |
| Negative events at Time 2 | 0.12 |  | 0.02 |  | .24 | *** | [.15, .32] |  |  |  | 0.11 |  | 0.02 |  | .22 | *** | [.13, .31] |  |  |
| Valuing happiness at Time 1 × Negative events at Time 2 | 0.00 |  | 0.00 |  | .01 |  | [–.07, .09] |  |  |  | 0.00 |  | 0.00 |  | .01 |  | [–.08, .09] |  |  |
| Depressive symptoms at Time 1 | – | | – | | – | | – |  |  |  | 0.02 |  | 0.02 |  | .06 |  | [–.04, .15] |  |  |
|  |  |  |  |  |  |  |  |  |  |  |  |  |  |  |  |  |  |  |  |
| Analysis 3: Interdependent self-construal as a moderator | | | | | | |  | .38 |  |  |  |  |  |  |  |  |  | .39 |  |
| Valuing happiness at Time 1 | 0.06 |  | 0.02 |  | .12 | * | [.03, .22] |  |  |  | 0.06 |  | 0.02 |  | .14 | ** | [.05, .23] |  |  |
| Reflection at Time 1 | 0.58 |  | 0.05 |  | .56 | *** | [.48, .63] |  |  |  | 0.54 |  | 0.05 |  | .52 | *** | [.43, .60] |  |  |
| Interdependent self-construal at Time 1 | 0.01 |  | 0.02 |  | .04 |  | [–.06, .14] |  |  |  | 0.00 |  | 0.02 |  | .00 |  | [–.10, .10] |  |  |
| Valuing happiness at Time 1 × Interdependent self-construal at Time 1 | 0.00 |  | 0.00 |  | .07 |  | [–.02, .16] |  |  |  | 0.00 |  | 0.00 |  | .07 |  | [–.02, .15] |  |  |
| Depressive symptoms at Time 1 | – | | – | | – | | – |  |  |  | 0.05 |  | 0.02 |  | .13 | ** | [.04, .23] |  |  |
| Note: Numbers in parentheses indicate 95% confidence intervals. * *p* < .05, ** *p* < .01, *** *p* < .001. | | | | | | | | | | | | | | | | | | | |
